# Endothelial Protease Activated Receptor 1 (PAR1) Signalling Is Required for Lymphocyte Transmigration across Brain Microvascular Endothelial Cells

**DOI:** 10.3390/cells9122723

**Published:** 2020-12-21

**Authors:** Silvia Dragoni, Anna Papageorgiou, Caroline Araiz, John Greenwood, Patric Turowski

**Affiliations:** Institute of Ophthalmology, University College London, 11-43 Bath Street, London EC1V 9EL, UK; s.dragoni@ucl.ac.uk (S.D.); annapapageorgiou@hotmail.com (A.P.); tonkacaro@yahoo.fr (C.A.); j.greenwood@ucl.ac.uk (J.G.)

**Keywords:** lymphocyte transendothelial migration, blood–brain barrier, protease-activated receptor 1, AMP-activated protein kinase, endothelial nitric oxide synthase

## Abstract

Lymphocyte transendothelial migration (TEM) relies on ICAM-1 engagement on the luminal surface of the endothelial cells (ECs). In blood–brain barrier (BBB) ECs, ICAM-1 triggers TEM signalling, including through JNK MAP kinase and AMP-activated protein kinase (AMPK), which lead to the phosphorylation and internalisation of the adherens junction protein VE-cadherin. In addition to ICAM-1, G protein-coupled receptors (GPCRs) are also required for lymphocytes TEM across BBB ECs. Here, we investigated the role of protease activated GPCRs (PARs) and found a specific role for PAR1 in support of lymphocyte TEM across BBB ECs in vitro. PAR1 requirement for TEM was confirmed using protease inhibitors, specific small molecule and peptide antagonists, function blocking antibodies and siRNA-mediated knockdown. In BBB ECs, PAR1 stimulation led to activation of signalling pathways essential to TEM; notably involving JNK and endothelial nitric oxide synthase (eNOS), with the latter downstream of AMPK. In turn, nitric oxide production through eNOS was essential for TEM by modulating VE-cadherin on Y731. Collectively, our data showed that non-canonical PAR1 activation by a lymphocyte-released serine protease is required for lymphocyte TEM across the BBB in vitro, and that this feeds into previously established ICAM-1-mediated endothelial TEM signalling pathways.

## 1. Introduction

Leukocyte extravasation from the blood stream to the interstitium is a critical step in the immune response. In vivo it can broadly be broken down into four distinct steps, namely chemoattraction, rolling adhesion, tight adhesion and finally TEM or diapedesis [1]. In vitro studies of TEM and associated signalling have greatly contributed to our current understanding of diapedesis, in particular how EC proactively guide leukocytes to adequate penetration sites and facilitate all transmigration steps between the luminal and abluminal side of the endothelium [2,3]. In our laboratories, we utilise a TEM-competent Th1 cell line, which induces experimental autoimmune encephalomyelitis (EAE, a model of multiple sclerosis) following adoptive transfer in vivo [4], to study TEM across resting and cytokine stimulated BBB ECs in vitro [2]. Using this model, we have previously shown that surface engagement of ICAM-1 by lymphocyte function-associated antigen 1 (LFA-1) is a rate limiting step for diapedesis of lymphocytes ECs [5], just as it is for TEM in other tissues [6]. ICAM-1 engagement leads to a plethora of EC signalling in BBB ECs, some of which is essential for subsequent TEM. Activation of SRC family kinases and rho GTPase lead to TEM-essential actin rearrangement [2,7]. AMPK, eNOS and JNK activation leads to VE-cadherin phosphorylation and internalisation to induce modulation of the paracellular space, presumably to render it TEM compliant [8,9,10]. In contrast, other ICAM-1-induced signalling such as through ERK and p38 shifts ECs gene expression to a more inflammatory phenotype [9]. In our model system of Th1 cell diapedesis across BBB ECs, signalling via heterotrimer G proteins is also required [11], indicating that the activation of endothelial surface GPCRs is similarly important than engagement of ICAM-1. GPCRs are the most diverse and largest group of membrane receptors in eukaryotes [12]. Topologically, GPCR polypeptide chains span membranes seven times, with the N-terminus exposed to the extracellular side of the membrane for extracellular ligand binding or sensing, and the C-terminus involved in the downstream signal generation through heterotrimeric G proteins. In the vascular system a wide variety of GPCRs regulate many critical functions ranging from vessel tone and leakage to chemokine sensing.

Protease-activated GPCRs are collectively known as protease-activated receptors (PARs), with its four members (PAR1-4) expressed in most mammalian cells [13]. Canonical activation of all PARs occurs via the irreversible cleavage of part of their extracellular N-terminus by a protease, most often a serine protease. This creates a tethered ligand leading to conformational change and activation of heterotrimeric G proteins. Subsequent signalling is tightly regulated by rapid desensitization at the plasma membrane and receptor trafficking [13,14]. PARs have different but overlapping expression patterns and are critical for coagulation and inflammatory responses. They are ubiquitously expressed throughout the vascular system. Importantly for the present study, brain ECs express all four PARs [15].

Activation of the same PAR can lead to vastly different downstream effects. This may depend on the cellular context but has also been clearly associated with ‘biased’ PAR signalling, in which different proteases cleave N-terminus at different positions to produce distinctive tethered ligands, inducing different conformational changes and consequently different activation of heterotrimeric G proteins [13,16].

PAR1, the major thrombin receptor in most cells, has important roles in several biological processes and can trigger distinct effects based on “biased” signalling [17]. For instance, in cerebral ECs, thrombin activation of PAR1 induces a pro-inflammatory phenotype, resulting in increased expression of ICAM-1 [18] and also barrier disruption [19], presumably through modulation of VE-cadherin [20]. By contrast, PAR1 activation by activated protein C (aPC) leads to S1P (sphingosine 1-phosphate) pathway-dependent barrier protective signalling [21,22]. It has also been shown that treatment with PAR1 antagonists attenuates the clinical symptoms of EAE in mice by reducing BBB breakdown [23]. Notable cellular effects in this study include suppression of the inflammatory response, down-regulation of matrix metalloproteinase-9 expression and preservation of the expression of occludin and zonula occludens (ZO)-1 in brain ECs.

For the present study, we hypothesized that PARs could be GPCRs required for lymphocyte TEM across BBB ECs. To investigate this, we measured TEM across resting, non-cytokine activated BBB ECs in the presence of protease inhibitors and following specific PAR neutralisation. From this, PAR1 emerged as a TEM GPCR. We therefore also investigated signalling downstream of PAR1 and its interconnection with established endothelial TEM signalling pathways.

## 2. Materials and Methods

### 2.1. Materials

PAR1 antibodies WEDE-15 and ATAP-2 were purchased from Beckman coulter (Fullerton, CA, USA) and Santa Cruz Biotechnologies (Santa Cruz, CA, USA), respectively. PAR2 antibody SAM-11 was purchased from Santa Cruz Biotechnologies. PAR1 agonist H-Thr-Phe-Leu-Leu-Arg-NH2 (TFLLR) and antagonist Mercaptopropionyl-Phe-Cha-Arg-Lys-Pro-Asn-Asp-Lys-NH2 (Mpr-NH2), PAR2 antagonist H-Phe-Ser-Leu-Leu-Arg-Tyr-NH2 (FSLLRY), and PAR4 antagonist trans-Cinnamoyl-Tyr-Pro-Gly-Lys-Phe-NH2 (tcY-NH2) were purchased from Peptides International (Louisville, KY). Anti-rat CD18 (clone WT3) and anti-rat CD11a (clone WT1) were from AbD Serotec (Kidlington, UK). Polyclonal Abs specific for phosphorylated forms of eNOS, p38, JNK, ERK1/2 (eNOS Ser1177, Erk1/2 Thr202/Tyr204, p38 Thr180/Tyr182 and SAPK/JNK Thr183/Tyr185) were from Cell Signaling Technology (Beverly, MA, USA); the anti-phosphotyrosine (4G10) was from Upstate Biotechnology, Millipore (Billerica, MA, USA), and the monoclonal antibody for tubulin was from Sigma-Aldrich (St Louis, MO, USA). Thrombin, Hirudin, BAPTA-AM, L-NAME (NG-nitro-L-arginine methyl ester), collagen IV and fibronectin were from Sigma-Aldrich. Compound C and antithrombin III were from Merck Biosciences (Nottingham, UK). SCH79797 was from Tocris (Bristol, UK).

### 2.2. Animals

Lewis female rats (6–8 weeks old) and C75BL/6J mice (7–12 weeks old) were purchased from Charles River Laboratories. All animal procedures were performed in accordance with Animal Welfare Ethical Review Body (AWERB) and Association for Research in Vision and Ophthalmology (ARVO) Statement for the Use of Animals in Ophthalmic and Vision Research guidelines and under a UK Home Office license.

### 2.3. Endothelial Cell Culture

The immortalized rat brain microvascular EC (BMVEC) line GPNT was cultured in F-10 medium supplemented with 10% FCS, 2 µg/mL bFGF, 80 µg/mL heparin, 100 i.u/mL penicillin, 100 µg/mL streptomycin on collagen 1–coated plastic-ware [24]. Primary cultures of cerebral microvascular ECs were prepared from 6–8-week-old Lewis rats or wild-type C57BL6 mice, as previously described [25]. Primary ECs were seeded on collagen IV/fibronectin-coated plates and maintained in EGM-2 MV (Lonza). Importantly, none of the treatments (pharmacological antagonists or transfections) had any effect on the EC monolayer integrity, which was routinely assessed by visual inspection and transendothelial electrical resistance (TEER) measurements.

### 2.4. Lymphocyte Coculture, Adhesion and Migration Assays In Vitro

**Figure 1:**
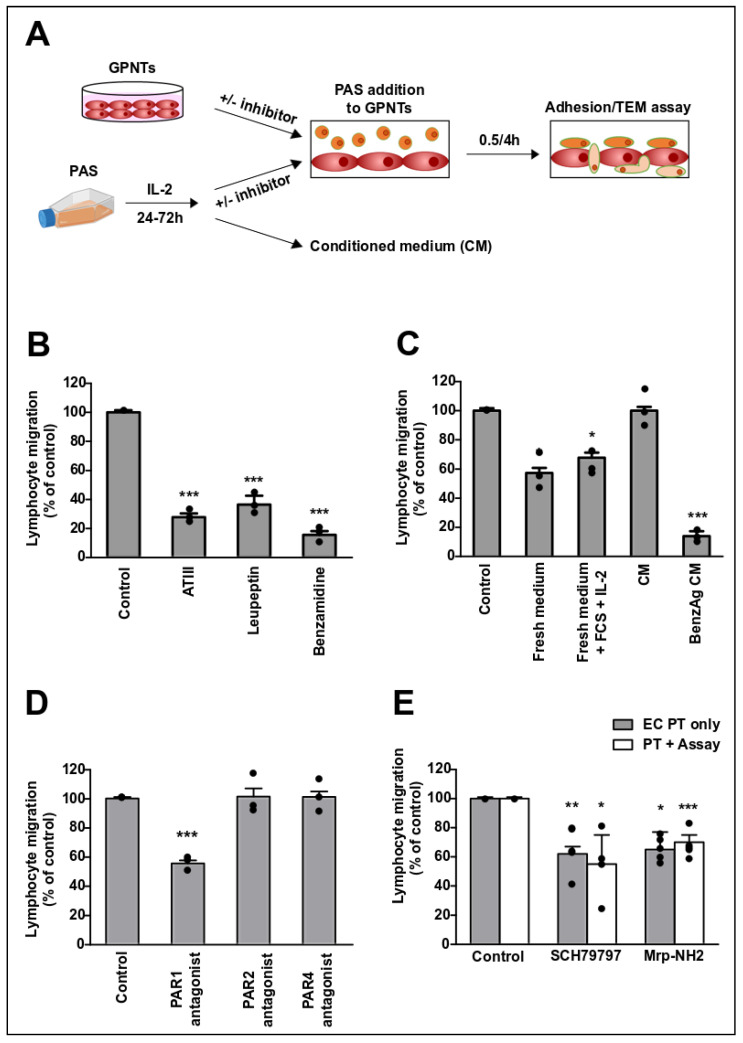
A T cell-secreted protease activates endothelial PAR1 during TEM. (**A**) Schematic of the experimental setup. GPNT blood–brain barrier (BBB) endothelial cells (ECs) were grown to confluence. PAS Th1 cells were activated in medium with IL-2 for 24–72 h. For some experiments the conditioned medium (CM) generated after 24 h was used separately. Optionally, GPNT or PAS were treated with inhibitors before PAS were added to the GPNT monolayer. Adhesion and TEM rates were assessed 0.5–4 h later. All PAS in contact with GPNT monolayers (apical, within, basal) were counted for adhesion. All PAS within and underneath were counted for TEM rates. (**B**) TEM assays were conducted in the presence of ATIII (1 U/mL containing 100 U/mL heparin), Leupeptin (2 µg/mL) or Benzamidine (1 mM) for 1h before PAS addition and migration assessment. (**C**) TEM assays were conducted as described in (**A**) for 4 h. Alternatively, PAS were collected by centrifugation after activation and resuspended before addition to GPNT for TEM assays in fresh culture medium (-FCS, -IL-2), fresh culture medium (+FCS, +IL-2), CM depleted of serine proteases using benzamidine-agarose. (**D**) GPNTs were pre-treated for 120 min with PAR1 (Mpr-NH2, 100 µM), PAR2 (FSLLRY, 400 µM) or PAR4 (tcY-NH2, 400 µM) peptide antagonists, before PAS were added and 4 h TEM rates were measured. (**E**) GPNTs were either pre-treated (PT) for 1 h or pre-treated and maintained (PT + Assay) during TEM with non-peptide (SCH79797, 1 µM) or peptide (Mrp-NH2, 100 µM) PAR1 antagonists. 4 h TEM rates are shown. Data represented as normalised means −/+ SEM. Each data point represents one independent, biological replicate. Significant differences from controls were determined by ANOVA and Dunnett’s post-hoc analysis with * *p* < 0.05, ** *p* < 0.01, *** *p* < 0.001.

The migratory Th1, myelin basic protein (MBP)-specific CD4+ lymphocyte cell line (PAS), which induces EAE by adoptive transfer in naïve rats [4], was cultured in RPMI-1640 supplemented with 10% FCS, 100 U/mL penicillin, 100 µg/mL streptomycin,1 mM sodium pyruvate, 1 mM nonessential amino acids, 2 mM L-Glu, and 50 µm β-mercaptoethanol. Prior to addition to BMVEC, recombinant IL-2 (50 U/mL) was added for 24–72 h, with longer IL-2 stimulation resulting in higher control TEM rates (see below). BMVECs monolayers in 96-well plates were co-cultured with IL-2 stimulated PAS cells, with each individual data point consisting of 6 individual culture wells (technical replicates). Additionally, inhibitors were added as described in Figure 1A and as specified in corresponding figure legends. After 30 min or 4 h, migration was assessed in each well using 5-min time-lapse microscopy [9]. Neutralization of LFA-1 was performed using function-blocking Abs against CD18 and CD11a [9]. Each TEM series was conducted as at least 3 biological replicates consisting of independently plated BMVECs and PAS stimulated with IL-2 for 24, 48 and 72 h. Average control TEM rates after 30 min were 6, 11 and 15% for 24, 48 or 72 h IL-2 stimulated PAS. After 4 h, these increased to 35, 42 and 61%, respectively. Data for each independent replicate series were normalised to control and then combined with the others for statistical analysis and graphical representation. Each independent experiment is represented as an individual data point in each figure.

Assays to measure lymphocyte adhesion to GPNT cells are based on radiolabel assays [7,11], which were modified for use with fluorescent cell labels as previously described [26]. Briefly, fluorescently labelled, concanavalin A (5 µg/mL)-activated rat peripheral lymph node (PLN) lymphocytes were added to GPNT monolayers, and after 90 min, adherent T cells were quantified in a fluorescent plate reader. Adhesion data was collected from triplicate experiments each consisting of 10 co-cultures. Control adhesion was 13–17.5% across all experiments.

### 2.5. RT-PCR

Total RNA from GPNTs was prepared using the RNeasy kit (Qiagen, Crawley, UK). 0.25 μg of total RNA was reverse transcribed using Superscript III (Invitrogen). PCR reactions were performed using 1 μg of cDNA and sequence-specific primers (see also Supplemental Figure S1A):

PAR1 (FWD 5′ CCT ATG AGA CAG CCA GAA TC 3′-REV 5′ GCT TCT TGA CCT TCA TCC 3′); PAR2 (FWD 5′ GCG TGG CTG CTG GGA GGT ATC 3′-REV 5′ GGA ACA GAA AGA CTC CAA TG 3′); PAR3 (FWR 5′ GTG TCT CTG CAC ACT TAG TG 3′-REV 5′ ATA GCA CAA TAC ATG TTG CC 3′); PAR4 (FWD 5′ GGA ATG CCA GAC GCC CAG CAT C 3′-REV 5′ GGT GAG GCG TTG ACC ACG CA 3′). PCR products were separated by agarose gel electrophoresis, stained with ethidium bromide, and acquired with GeneSys software (Syngene). The molecular weight of the PCR product was compared with the 50 bp DNA ladder (New England BioLabs, Hitchin, UK). Identity of PCR products was verified by additional restriction enzyme digests and DNA sequencing.

### 2.6. siRNA Knockdown of PAR1

GPNTs were transfected with targeting siRNA as previously described [8]. Briefly, sub-confluent GPNTs were transfected using oligofectamine reagent (Invitrogen, Paisley, UK). Targeting PAR1 siRNA duplexes (200 nM) and non-targeting controls (Dharmacon, Chicago, IL, USA) were transfected in serum-free medium for 4 h, before serum was added back into the medium. After an overnight incubation, the transfection was repeated, and 72 h after the first transfection, the migration assay, as well as the western blotting for PAR-1 protein knockdown (using ATAP-2 antibody), were performed.

### 2.7. Immunoblotting

Cell extracts were prepared by lysis in boiling 50 mM Tris/Cl, pH 6.8, 2% SDS, 10% glycerol, 100 mM DTT. Proteins were separated by SDS-PAGE and transferred to nitrocellulose by semidry electrotransfer. Membranes were blocked o/n and then incubated with the appropriate antibody diluted at 1:2000. Membranes were washed three times with TBS/0.1% Tween-20 before 1h incubation with an anti-mouse or anti-rabbit HRP-conjugated IgG (GE Healthcare) at a dilution of 1:10,000 and 1:5000, respectively. Membranes were developed using the ECL reagents (Roche) and exposed to X-ray film. Protein bands were evaluated by densitometric quantification using the NIH imaging software ImageJ and normalized against the amount of total protein and tubulin.

### 2.8. VE-Cadherin Plasmids

Mouse VE-cadherin-EGFP expression plasmids (pEGFP-N1-mVEC) were used for exogenous expression of wild type VE-cadherin in GPNT cells as described26. The Y731 to E substitution was introduced by Quickchange mutagenesis (Stratagene) using the oligonucleotides mVEC-Y731E-up

(5′ ACGACACACTGCACATCGAGGGATACGAGGGCGCAGAGTCCA 3′)

and mVEC-Y731E-low

(5′ TGGACTCTGCGCCCTCGTATCCCTCGATGTGCAGTGTGTCGT 3′).

All plasmids were verified by DNA sequencing and purified using endotoxin-free preparation methods (Qiagen) before nucleofection (Amaxa) into GPNT cells.

### 2.9. Data Analysis and Statistics

Data are presented as mean ± SEM. TEM and adhesion data were expressed as percentage of control (TEM: mean ± SEM of six replicates from at least three independent experiments; adhesion: mean ± SEM of six replicates from three independent experiments). Densitometric quantifications of four independent immunoblots were determined by changes in phosphoprotein content normalized to tubulin and total protein loading controls, with values expressed as fold increase. Statistics were performed using one-way ANOVA, with significance levels set at 0.05, followed by Dunnett’s or Tukey’s post-hoc tests. Alternatively, Student t test was used for pairwise comparison. * *p* < 0.05; ** *p* < 0.01; *** *p* < 0.001.

## 3. Results

### 3.1. Endothelial PAR-1 Is Required for Lymphocyte Migration across Rat Brain Microvascular ECs

Our experimental system to study lymphocyte migration across brain microvascular ECs consisted of rat GPNT endothelial cell monolayers incubated apically with PAS cells, a TEM competent MBP-specific rat Th1 cell line [4,9] (Figure 1A). Prior to their addition to endothelial cell monolayers, PAS cells were activated for at least 24 h in medium containing IL-2 as detailed in the Method section. To investigate if PARs are among GPCRs required for PAS TEM across GPNT, we measured migration rates in the absence and presence of broad specificity protease inhibitors. Antithrombin III (ATIII), leupeptin and benzamidine significantly reduced TEM by at least 60% (Figure 1B). Inhibition by benzamidine was strongest and, by reducing TEM to less than 20%, in the range that is observed following LFA1-ICAM-1 blockade [5] (see also Figure 1E) or GPCR inhibition [11].

When activated PAS were resuspended in fresh medium either with or without IL-2 prior to TEM assays, migration rates were reduced by ca. 40% (Figure 1C). Full TEM rates were restored when PAS were resuspended in 24 h pre-conditioned medium. However, when activated PAS were resuspended in 24 h pre-conditioned medium depleted of serine protease, PAS transmigration rates were reduced to <20%, similar to what was seen when benzamidine was present throughout migration. Taken together, this indicated that serine protease(s) secreted by the lymphocytes were essential for TEM of PAS across GPNT BBB ECs.

Serine proteases induce cellular responses via PAR1-4 [13]. The expression of all four PARs was detected in GPNT cells by RT-PCR (Supplemental Figure S1) in agreement with published gene expression [15]. Of the many canonical and non-canonical proteases that can activate PAR1-4 [13,27,28], only activated protein C, trypsin, MMPs, granzymes, chymase, kallikreins, calpains, cathepsin, matripase and testisin are expressed by CD4+ lymphocytes (Table 1). Amongst these, only activated protein C and trypsin can activate PAR3. However, since trypsin is inactivated by FCS, and protein C requires a complex activation cascade not present in tissue culture, the PAS Th1 cell-secreted protease(s) in our co-cultures could potentially activate PAR1, 2 and 4 but not PAR3.

**Figure 2:**
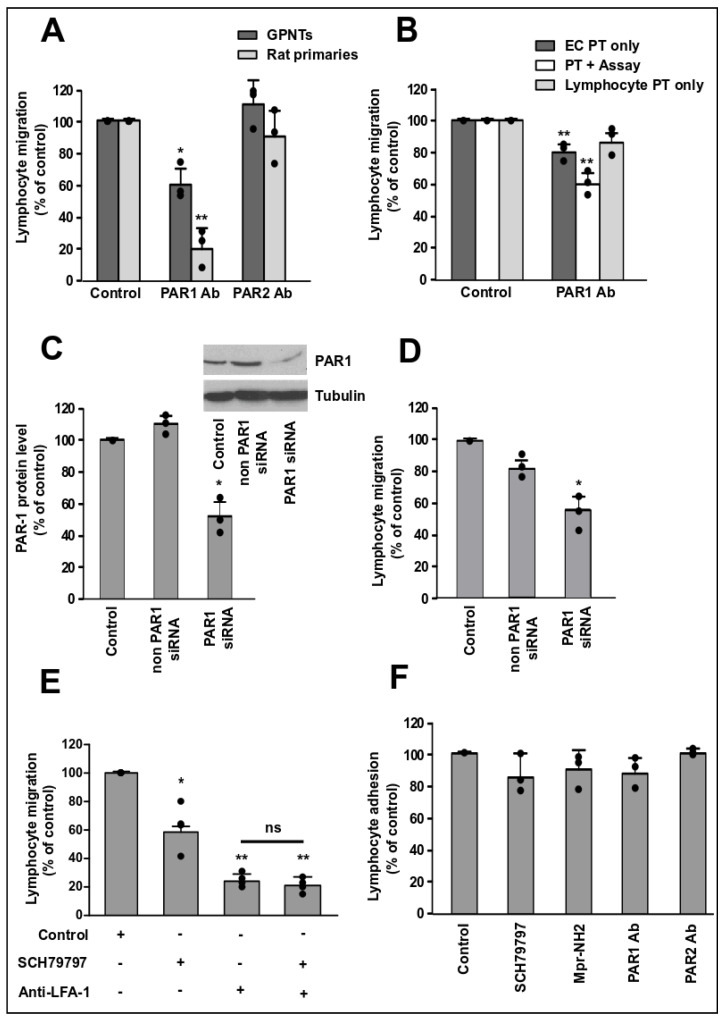
Endothelial PAR1 is required for TEM. (**A**) GPNT or primary rat BMVEC were pre-treated for 1 h and maintained during the TEM assay with PAR blocking antibodies (ATAP-2, 10 µg/mL for PAR1; SAM-11, 30 µg/mL for PAR2). PAS lymphocyte migration was then measured after 4 h. (**B**) TEM rates were assessed after 30 min to eliminate effects due to altered gene expression. GPNT were either pre-treated (EC PT) or pre-treated and maintained (PT + assay) with the PAR1 blocking antibody ATAP-2 (10 µg/mL). Alternatively, only PAS were pre-treated (Lymphocyte PT). (**C**) GPNTs were transfected with siRNA non-targeting or targeting PAR1 (200 nM) and PAR1 levels were assessed in relation to cellular tubulin by western blot (representative example shown in the inset). Densitometric quantification of 3 independent experiment showed that knock-down led to reduction of PAR1 by ca. 50%. (**D**) 4 h TEM rates across siRNA transfected GPNT. (**E**) GPNTs were pre-treated with PAR1 antagonist (SCH79797, 1 µM). Alternatively, LFA-1 on PAS was neutralised by pre-incubation with anti-CD18 and CD11a before 4 h TEM rates were measured. In addition, TEM rates following combination of both pre-treatments were assessed. (**F**) T cell adhesion to GPNT treated with the indicated compounds for 1 h before and throughout the adhesion assay. Data represented as normalised means −/+ SEM. Each data point represents one independent, biological replicate. Significant differences from controls were determined by ANOVA and Dunnett’s or Tukey’s post-hoc analysis with * *p* < 0.05, ** *p* < 0.01.

The peptide-based PAR1 antagonist Mpr-NH2 significantly inhibited TEM (Figure 1D). Similar peptide antagonists targeting PAR2 or PAR4 did not affect TEM at all, even at much higher concentrations. PAS migration was similarly affected by the peptide PAR1 antagonist Mrp-NH2 and the non-peptide PAR1 antagonist SCH79797 (Figure 2E). Importantly, both were equally effective when used only in EC pre-treatment or when also maintained throughout the TEM assay.

The requirement for PAR1 for PAS TEM across GPNT was further confirmed using PAR1 specific antibodies, which, when used to pre-treat GPNT and included during the TEM assays, reduced PAS migration rates by ca. 40% (Figure 2A). In clear contrast, antibodies inhibiting PAR2 did not affect TEM. Importantly, even stronger inhibition of PAS TEM by PAR1 antibodies was observed when TEM was measured across primary rat brain ECs rather than GPNT cells, indicating that dependence on PAR1 was not specific to the GPNT cell line but a general characteristic of cerebral ECs.

Endothelial PAR1 activation can induce inflammatory gene expression [18], which may stimulate PAS TEM. However, initial TEM that occurs within 30 min is unaffected by gene expression changes [9]. To separate effects of PAR1 activation on diapedesis from those on transcription, shorter TEM assays of only 30 min were also conducted. Three regimens of PAR1 antibody blockade were used: (1) pre-treatment only the endothelial monolayer, (2) pre-treatment of the endothelial monolayer and maintenance of antibody throughout the migration assay, and (3) pre-treatment only of the PAS lymphocytes (Figure 2B). Whilst lymphocyte pre-treatment did not affect 30 min TEM rates significantly, EC pre-treatment with PAR-1 reduced TEM rates to 80% and further to 60% when the blocking antibody was maintained throughout the assay. Overall, this indicated that blockade of PAR1 on ECs but not lymphocytes directly affected diapedesis. It also indicated that ECs were the cells relying on PAR1 activation during TEM.

Specific knock-down of PAR1 using siRNA also significantly affected PAS migration across GPNTs to similar extent than PAR1 antagonists or antibodies (Figure 2C,D). Overall, PAR1 antagonism led to less TEM inhibition than what was seen by LFA-1 blockade (Figure 2E). Notably, PAR1 and LFA-1 blockade did not produce additive inhibition of TEM, suggesting that both affected the same downstream signalling pathways.

Importantly, PAR1 or PAR2 neutralisation by any of the treatments mentioned above did not significantly affect lymphocyte adhesion (Figure 2F), indicating that only diapedesis was affected by PAR1 inhibition.

**Figure 3:**
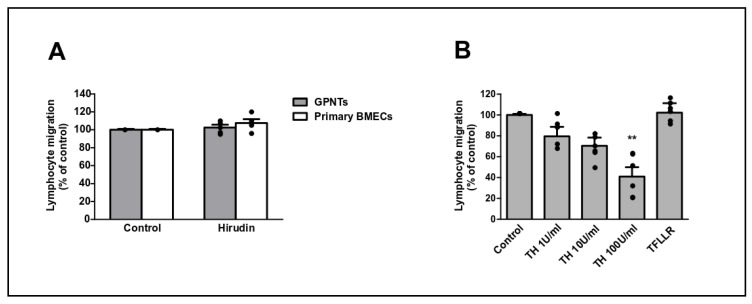
Thrombin does not activate TEM. (**A**) GPNT or primary BMVECs and PAS were pre-treated for 1 h with Hirudin (20 U/mL) and then combined for 4 h TEM assays. (**B**) GPNT were pre-treated for 1h with the indicated concentrations of thrombin or the PAR-1 TFLLR agonist before PAS were added and 4 h TEM assessed. Data represented as normalised means −/+ SEM. Each data point represents one independent, biological replicate. Significant differences from controls were determined ANOVA and Dunnett’s post-hoc analysis with ** *p* < 0.01 (vs. control).

In many cell systems, the main PAR1 activator is thrombin. Indeed, thrombin strongly affects ECs including their barrier function by inducing paracellular permeability [19]. However, thrombin is not expressed by lymphocytes (Table 1). Accordingly, we were unable to detect prothrombin gene expression in GPNT or PAS cells or measure any thrombin activity in PAS conditioned medium or in medium of PAS-GPNT co-cultures. Furthermore, the specific thrombin inhibitor hirudin did not affect PAS TEM across GPNT (Figure 3A). Pre-treatment of GPNT for 30 min with thrombin resulted in dose dependent inhibition of TEM (Figure 3B), indicating proteolytic desensitisation of the PAR required for TEM. This desensitisation could not be reproduced by the PAR1-specific peptide agonist TFLLR.

### 3.2. PAR1 Activation Leads to MAPK, AMPK and eNOS Activation in Brain Microvascular ECs

**Figure 4:**
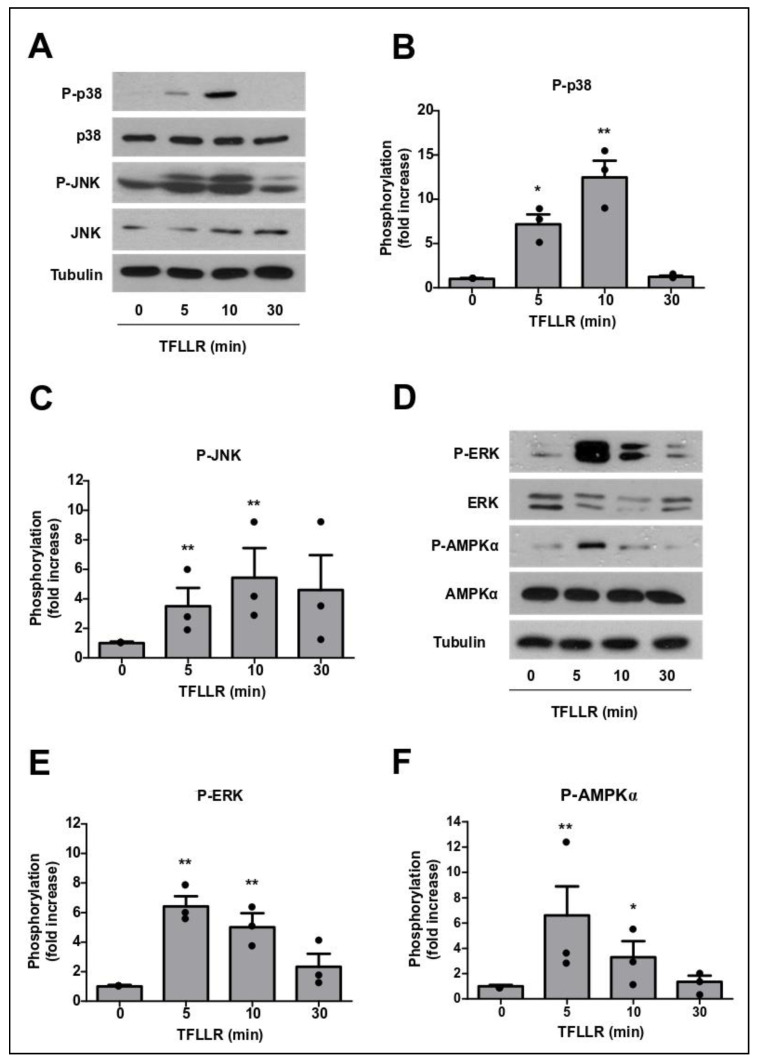
PAR1 activation in BBB endothelial cells leads to MAPK and AMPK phosphorylation. GPNT were stimulated with 100 µM TFLLR for the indicated length of time, and p38 (**A**,**B**), JNK (**A**,**C**), ERK (**D**,**E**) and AMPKα (**D**,**F**) phosphorylation was assessed by western blot. Representative results and quantification of protein phosphorylation, normalised to total protein levels, from three independent experiments are shown as means ± SEM. Each data point represents one independent, biological replicate. Significant differences from controls were determined by ANOVA and Dunnett’s post-hoc analysis with * *p* < 0.05, ** *p* < 0.01.

To study PAR1 downstream signalling, we used the PAR1-specific peptide TFLLR, which mimics PAR1 signalling more broadly than thrombin [13]. The addition of TFLLR to GPNT monocultures led to rapid and transient induction of protein kinases previously activated by ICAM-1 ligation [8,9]. Specifically, we observed phosphorylation of p38 MAPK, which reached a peak after 10 min, returning to control levels by 30 min (Figure 4A,B). JNK MAPK phosphorylation was also significantly increased in response to TFLLR, with a time course similar to p38 (Figure 4A,C). ERK MAPK phosphorylation reached a peak earlier, at 5 min, returning to control levels at 30 min (Figure 4D,E). Similarly, AMPK was strongly phosphorylated in response to TFLLR, with a transient peak at 5 min (Figure 4D,F).

**Figure 5:**
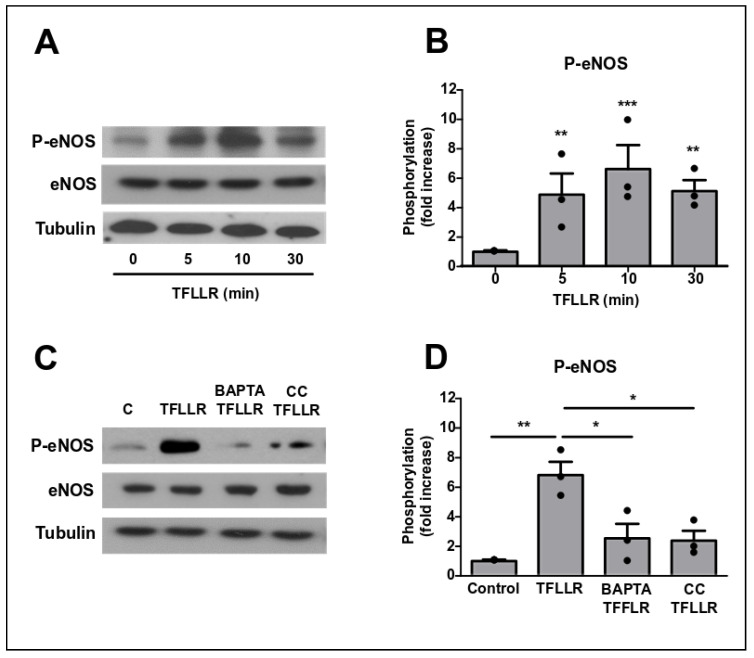
PAR1 activation in BBB endothelial cells leads to eNOS activation. (**A**,**B**) GPNT were stimulated with 100 µM TFLLR for the indicated length of time before eNOS phosphorylation was analysed by western blot. (**C**,**D**) GPNT were incubated with BAPTA (20 µM) or Compound C (10 µM) for 15 min and then treated with TFLLR for 5 min. Phosphorylation of eNOS was assessed by western blot. Representative results and quantification of protein phosphorylation, normalised to total protein levels, from three independent experiments are shown as normalised means ± SEM. Each data point represents one independent, biological replicate. Significant differences from controls were determined by ANOVA and Dunnett’s and Tukey’s post-hoc analysis with * *p* < 0.05, ** *p* < 0.01, *** *p* < 0.001.

Endothelial Nitric oxide synthase (eNOS), which is an important mediator of ICAM-1-mediated TEM signalling [8], was also activated by TFLLR (Figure 5A,B). Its phosphorylation on S1179 followed a similar time course to that of p38, JNK and AMPK with a peak at 10 min following TFFLR addition. Phosphorylation was sensitive to Ca^2+^ chelation using BAPTA and AMPK inhibition using compound C (CC) (Figure 4C,D) and was in that regard similar to what has been described its activation downstream of ICAM-1 engagement [8].

### 3.3. Transendothelial Migration Requires Ca^2+^, AMPK, eNOS and VEC

**Figure 6:**
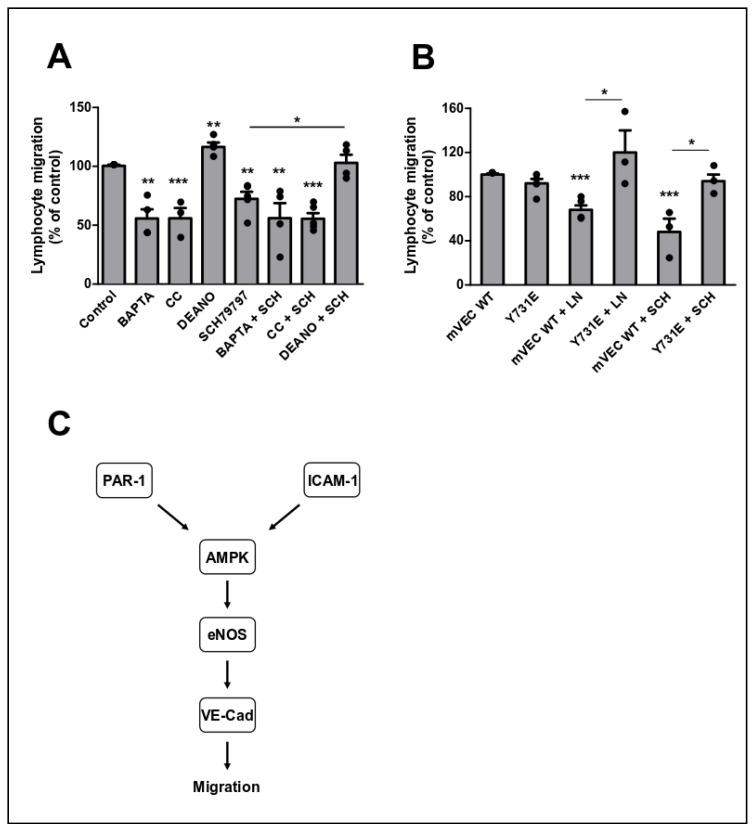
NO and VE-cadherin are downstream of PAR1 in TEM-regulating endothelial signalling. (**A**) GPNT were pre-treated for 2 h and maintained with BAPTA (20 μM), compound C (CC, 10 μM), DEANO (50 μM) or SCH79797 (1 μM) and the indicated compound combinations. PAS were added and TEM was assessed 4 h. (**B**) GPNT were transfected with either pEGFP-N’-mVEC WT or Y731E encoding wild type mouse VE-cadherin or the phosphomimetic mutant Y731E and grown to confluency for 24–48 h. GPNTs were then either left untreated (mVEC WT, Y731E) or pre-treated with and maintained in PAR1 non-peptide antagonist SCH79797 (SCH, 1 µM) for 2 h. L-NAME pre-treatment (LN, 1 mM) was administered 1 h before PAS were added to the monolayer. Data represented as normalised means −/+ SEM. Each data point represents one independent, biological replicate. Significant differences were determined by ANOVA and Tukey’s post-hoc analysis (v control or pairwise as indicated). * *p* < 0.05, ** *p* < 0.01, *** *p* < 0.001. (**C**) Proposed signalling network downstream of PAR-1 and ICAM-1.

Next, we studied the involvement of the PAR1-AMPK-eNOS signalling cascade in TEM in more detail (Figure 6A). BAPTA and CC reduced PAS TEM to levels previously reported [8]. TEM inhibition using the PAR1 antagonist SCH79797 was not reduced further by co-administration of BAPTA or CC, indicating that the same signalling pathway was affected. The addition of the NO donor DEANO, alleviating the requirement for eNOS activity, abolished the inhibitory effect of SCH79797, demonstrating the requirement for NO in TEM-specific PAR1 signalling.

VE-cadherin phosphorylation is an important endpoint in TEM signalling [15,29]. Specifically, mutation of Y731 to F731 leads to strong inhibition of PAS migration across GPNT [26]. VE-cadherin phosphorylation is also dependent on eNOS activation, at least with regards to signalling downstream of ICAM-1 [8]. Here, we investigated the eNOS to VE-cadherin Y731 signalling with respect to PAS TEM and in particular also PAR1 signalling. For this, we used plasmids expressing either wt VE-cadherin or VE-cadherin carrying a Y731E mutation, introduced to mimic tyrosine phosphorylation. Exogenous expression of either VE-cadherin did not affect baseline migration rates (Figure 6B). When TEM was blocked using the NOS inhibitor L-NAME, TEM was inhibited in the presence of wt but not Y731E VE-cadherin, indicating that the Y731E mutation compensated for loss of eNOS activity. Similarly, TEM reduction in response to PAR1 antagonism was completely reversed in the presence of Y731E but not wt VE-cadherin, suggesting that VE-cadherin and its phosphorylation on Y731 was an important endpoint of PAR1 TEM signalling.

## 4. Discussion

Many secreted or cell surface proteases support a wide variety of leukocyte function including the destruction of pathogens (such as by neutrophil elastase) [30], protection from malignant target cells (such as granzymes), interstitial migration (such as by cathepsin X) or chemokine processing (such as by caspase-1) [31]. Here, we demonstrated that a CD4+ T cell line secreted a protease activity that activated endothelial PAR1 and thereby supported its TEM across resting BMVECs. To our knowledge, this is the first report assigning such function to a leukocyte protease.

PAS cells are antigen activated and require stimulation with IL-2 to become migratory. Our results showed that during the IL-2 stimulation in vitro, these Th1 cells produced a TEM stimulating activity, which was sensitive to serine protease inhibitors and could be eliminated by absorption to benzamidine-agarose. Investigation of published gene expression data and correlation with ATIII sensitivity indicated that Th1 cells express proteases that can activate all four PARs. However, none of the potential PAR3 activating proteases could be active in our experimental system. Involvement of PAR2 and PAR4 was excluded through specific antagonist studies. This left only PAR1, and its role in supporting TEM was confirmed by antagonists, antibodies and siRNA. Since PAR1 is also expressed by T cells, it is conceivable that autocrine activation of the T cell is also important for TEM. However, we found no significant evidence for this in our assays (Figure 2B) and thus only focused on how PAR1 in BBB BMVECs could contribute to T cell transmigration. Importantly, the only likely TEM-stimulating proteases activating PAR1 in our experimental setup could be granzymes, chymase and kallikreins (Table 1). All three proteases may conceivably play a role in lymphocyte TEM: Granzymes are abundantly present during tissue remodelling in chronic inflammation [32]. Classically, they are recognised to play a key role in the induction of lymphocyte-induced cell death including that of neurons during neuroinflammation [33]. Notably, granzyme B inhibition leads to neuronal protection and attenuation of EAE [34]. More subtle roles, not leading to cell death, have been identified for granzmes, many of which affect the cardiovascular system. These include the induction of vascular dysfunction and leakage [35]. Chymase is most strongly expressed in mast cells, in which it is a major constituent of secretory granules [36]. Whilst our review of expression data did not demonstrate unambiguous expression in lymphocytes, chymase levels are correlated with levels of tumour-infiltrating lymphocytes, suggesting the potential of this neutral serine protease to enhance lymphocyte migration. Lastly, whilst kallikrein expression in lymphocytes is not unambiguous, the kallikrein-kinin protease system has a demonstrated role during immune-cell infiltration across the BBB in EAE. Plasma kallikrein, via endothelial PAR2, leads to upregulation of cellular adhesion molecules and subsequent enhanced leukocyte trafficking [37]. Activation of the kinin receptor B1 also reduces lymphocyte TEM across BBB ECs and their infiltration into the CNS during EAE [38]. None of these prior studies point to a direct role of these protease in TEM across the BBB, though. Importantly, granzymes, chymase and kallikreins activate PAR1 by non-canonical cleavage, with the cleavage sites yet to be identified [13]. Thus, it is technically very difficult to identify the proteolytic site and the tethered ligand. Whilst it will be interesting to elucidate which lymphocyte protease indeed activates PAR1 during TEM, our focus here was on understanding the role played by PAR1 signalling in the ECs.

PAR1 regulates a wide variety of cardiovascular—often clearly proinflammatory—functions [39]. Prominent amongst these are platelet and EC activation resulting in the release of clotting factors. In endothelial cells, enhanced inflammatory gene expression including that of adhesion molecules such as ICAM-1 and VCAM-1 is typically seen in response to PAR1 activation. Typically, disruption of endothelial barrier and vascular leakage are also a consequence of PAR1 activation, although thrombin and activated protein C have opposing effects on EC barrier function [19,22]. Given that most PAR1 functions have been studied using the canonical activator thrombin, many different functions based on biased PAR1 signalling may yet be discovered. Thrombin also induces permeability in cerebral microvascular ECs and compromises the integrity of the BBB during head trauma, ischemic stroke, inflammation and neurodegeneration [19]. However, thrombin was clearly not involved in PAR1 signalling relevant to TEM. Hirudin, which is monospecific for thrombin did not affect TEM. Furthermore, thrombin addition to TEM co-cultures led to inhibition of TEM, strongly indicating that PAR1 desensitisation had occurred. Notably, TFLLR did not induce similar desensitisation. Since both thrombin cleavage and TFFLR binding lead to PAR1 internalisation, thrombin desensitisation was likely due to removal of the TEM-specific protease cleavage site and a resulting disarming of the receptor, similar to the previously described biased PAR1 activation in response to neutrophil elastase and proteinase-3 [40].

TFFLR, which has been used to model PAR1 more broadly [13], induced endothelial signalling previously associated with ICAM-1 surface engagement. Endothelial ERK and p38 MAPK activation following exposure of GPNTs to PAS cells is not required for immediate TEM but shifts EC gene expression to a more inflammatory pattern and may enhance subsequent leukocyte recruitment [9]. PAR1 is likely to reinforce pro-inflammatory gene expression. By contrast, activation of JNK and AMPK influences TEM rates directly with VE-cadherin phosphorylation and internalisation one important downstream effector process [8,9]. Similar to what is observed in response to ICAM-1 engagement, activation of eNOS was yet another hallmark of TFFLR, with both Ca^2+^ and AMPK upstream of its activation. This indicated that to support lymphocyte TEM, PAR1 activation fed at least in part into the same endothelial signalling pathways than ICAM-1 engagement, hierarchically involving Ca^2+^, AMPK, eNOS and VE-cadherin (Figure 6C). That PAR1 and ICAM-1 activated the same pathways was also supported by our observations that inhibition of PAR1 and ICAM-1 activation did not produce additive TEM reduction. VE-cadherin was yet again confirmed as a molecular endpoint of TEM. Indeed, it was noteworthy that expression of a Y731E mutant of VE-cadherin completely attenuated the requirement for PAR1 activation during TEM. Originally, it was shown that the phosphorylation of VE-cadherin on Y731 is required for TEM of lymphocytes and neutrophils [26,41]. More recently it has been suggested that it is rather subsequent dephosphorylation of this site that drives TEM [15,29]. Whilst our Y731E mutant may not have replicated the phosphorylation-dephosphorylation cycle accurately, it nevertheless established a robust molecular link between PAR1 and VE-cadherin in the context of TEM signalling.

Serine protease inhibition using AT III or benzamidine resulted in strong, ca. 80% reduction of TEM, comparable to what is achieved in GPNT cells by heterotrimeric G protein inhibition using pertussis toxin [11] or LFA-1/ICAM-1 blockade (Figure 1E) [5]. However, selective inhibition of PAR1 only resulted ca. 40–50% reduction in TEM across GPNT (albeit by more when using primary cerebral ECs, which are generally more sensitive to TEM inhibitors), and this irrespective of the way PAR1 was neutralised (antagonists, antibodies, siRNA). This indicated that proteases beyond those activating PARs may play an additional role in TEM.

In conclusion, our data identified biased PAR1 signalling in BBB ECs as an important molecular requirement for lymphocyte TEM. Crucially, PAR1 signalling overlapped with that induced with ICAM-1. Future work should focus on whether PAR1 is generally required for leukocyte migration across any vascular bed, on whether PAR1 also induces endothelial signalling pathways different from those activated by ICAM-1, and on the identification of the PAR1-activating protease during TEM.

## Figures and Tables

**Table 1:** Literature was searched for expression of PAR1-4 activating proteases in CD4+ cells as well as their inhibition by ATIII. Proteases, for which no evidence of CD4+ cell expression could be found, are in grey. Note that AT III inhibited PAS TEM (Figure 1B). Thus, only CD4+ expressed proteases that are sensitive to AT III are likely candidates of PAR activation in PAS-GPNT co-cultures. For PAR1 activating proteases, only granzymes, chymase and kallikreins (highlighted in green) fitted these criteria.

	Activating Protease (*gene*)	Expression in T Cells		Inhibition by ATIII ^‡^
		hCD4+ (Th1) *	mCD4+ ^†^	
**PAR1**	Thrombin^13, 27, 28^ (*f2*)	no	no	
	Activated Protein C^13, 27, 28^ (*proc*)	very low	no	weak, but ^§^
	Factor VIIa^13^ (*f7*)	no	no	
	Factor Xa^13, 27, 28^ (*f10*)	no	no	
	Trypsin^13, 28^ (*prss1,2,3*)	very low (*prss1*)	yes (*prss2*)	yes, but ^¶^
	Plasmin^13, 27, 28^ (*plg*)	no	no	
	MMPs^13, 27, 28^ (*mmp1,2,3,8,9,12,13*)	yes (*mmp9*)	yes (*mmp1,2,8,9*)	no
	Neutrophil Elastase^13, 27^ (*elane*)	no	no	
	Proteinase-3^13, 27^ (*prtn3*)	no	no	
	Granzymes^13, 27, 28^ (*gzma,gzmb,gzmk*)	high	yes	yes
	Chymase^13^ (*cma1*)	no	yes	yes ^#^
	Cathepsin G^13^ (*ctsg*)	no	no	
	Kallikreins^13^ (*klk4,5,6,14*)	yes (*klk14*)	no	yes
	Calpain-1^13^ (*capn1*)	high	yes	no **
**PAR2**	Trypsin^13, 27, 28^ (*prss1,2,3*)	very low (*prss1*)	yes (*prss2*)	yes, but ^¶^
	Tryptase^13, 27, 28^ (*tpsab1*)	no	no	
	Factor VIIa^27, 28^ (*f7*)	no	no	
	Factor Xa^27, 28^ (*f10*)	no	no	
	Kallikreins^13, 27, 28^ (*klk4,5,6,14*)	yes (*klk14*)	no	yes
	Neutrophil Elastase^13, 27^ (*elane*)	no	no	
	Proteinase-3^13, 27^ (*prtn3*)	no	no	
	Cathepsins^13, 27^ (*ctss, ctsg*)	high (*ctss*)	yes (*ctss*)	n/d
	Granzyme A^28^ (*gzma*)	high	yes	yes
	Matripase^28^ (*st14*)	no	yes	yes
	Thrombin^13^ (*f2*)	no	no	
	Activated Protein C^13^ (*proc*)	very low	no	weak, but ^§^
	Chymase^13^ (*cma1*)	no	yes	yes
	Plasmin^13^ (*plg*)	no	no	
	Testisin^13^ (*prss21*)	yes	no	n/d
	Calpain-2^13^ (*capn2*)	high	yes	no **
**PAR3**	Thrombin^13, 27, 28^ (*f2*)	no	no	
	Activated Protein C^13, 27^ (*proc*)	very low	no	weak, but ^§^
	Factor Xa^13^ (*f10*)	no	no	
	Trypsin^13^ (*prss1,2,3*)	very low (*prss1*)	yes (*prss2*)	yes, but ^¶^
**PAR4**	Thrombin^13, 27, 28^ (*f2*)	no	no	
	Trypsin^13, 27, 28^ (*prss1,2,3*)	very low (*prss1*)	yes (*prss2*)	yes, but ^¶^
	Factor Xa^28^ (*f10*)	no	no	
	Plasmin^27, 28^ (*plg*)	no	no	
	Cathepsin G^13, 27, 28^ (*ctsg*)	no	no	
	MASP1^27^ (*masp1*)	no	no	
	Kallikrein14^13, 28^ (*klk14*)	yes	no	yes

* Gene expression in human CD4+ (Th1) cells was verified using the DICE database (https://dice-database.org). Very low expression defined as <1 transcript/million. ^†^ Gene expression in mouse CD4+ cells was verified using the databrowsers on ImmGen and the DeepRNA dataset (http://www.immgen.org). ^‡^ Literature was searched for evidence of inhibition. Only proteases, for which evidence of expression in CD4+ could be found, were investigated. ^§^ Protein C is a zymogen, which requires activation through a cascade involving thrombin and protein S, which are unlikely to be present in our system. ^¶^ Trypsin could not be active in PAS-GPNT co-cultures due to the presence of FCS. ^#^ To our knowledge, chymase interaction with ATIII has not been studied. However, chymase being a chymotrypsin-like enzyme is likely to be inactivated by ATIII. ** To our knowledge, ATIII inhibition of calpains has not been studied. However, calpains are cysteine proteases and usually not inhibited by serpins. n/d. To our knowledge not determined. For the present study not relevant as not activating PAR1. Superscript numbers represent the references linking the listed protease to respective PARs.

## Supplementary Materials

The following are available online at https://www.mdpi.com/2073-4409/9/12/2723/s1. Figure S1: All four PARs were detected in GPNT cells.

## Abbreviations

AMPK	AMP-activated protein kinase
aPC	activated protein C
ATIII	anti-thrombin III
BBB	blood brain barrier
BMVEC	brain microvascular endothelial cell
EAE	experimental autoimmune encephalomyelitis
EC	endothelial cell
eNOS	endothelial nitric oxide synthase
GPCR	G protein-coupled receptor
LFA-1	lymphocyte function-associated antigen 1
Mpr-NH2	Mercaptopropionyl-Phe-Cha-Arg-Lys-Pro-Asn-Asp-Lys-NH2
tcY-NH2	trans-Cinnamoyl-Tyr-Pro-Gly-Lys-Phe-NH2
PAR	protease-activated receptor
PLN	peripheral lymph node
S1P	sphingosine 1-phosphate
TEER	transendothelial electrical resistance
TEM	transendothelial migration
