# Supplementary material for: Endothelial Protease Activated Receptor 1 (PAR1) Signalling Is Required for Lymphocyte Transmigration across Brain Microvascular Endothelial Cells
Source: Cells. 2020 Dec 21;9(12):2723. doi: 10.3390/cells9122723 (PMC7766634; doi:10.3390/cells9122723)
Supplement: Supplementary file 1 [file cells-09-02723-s001.pdf]

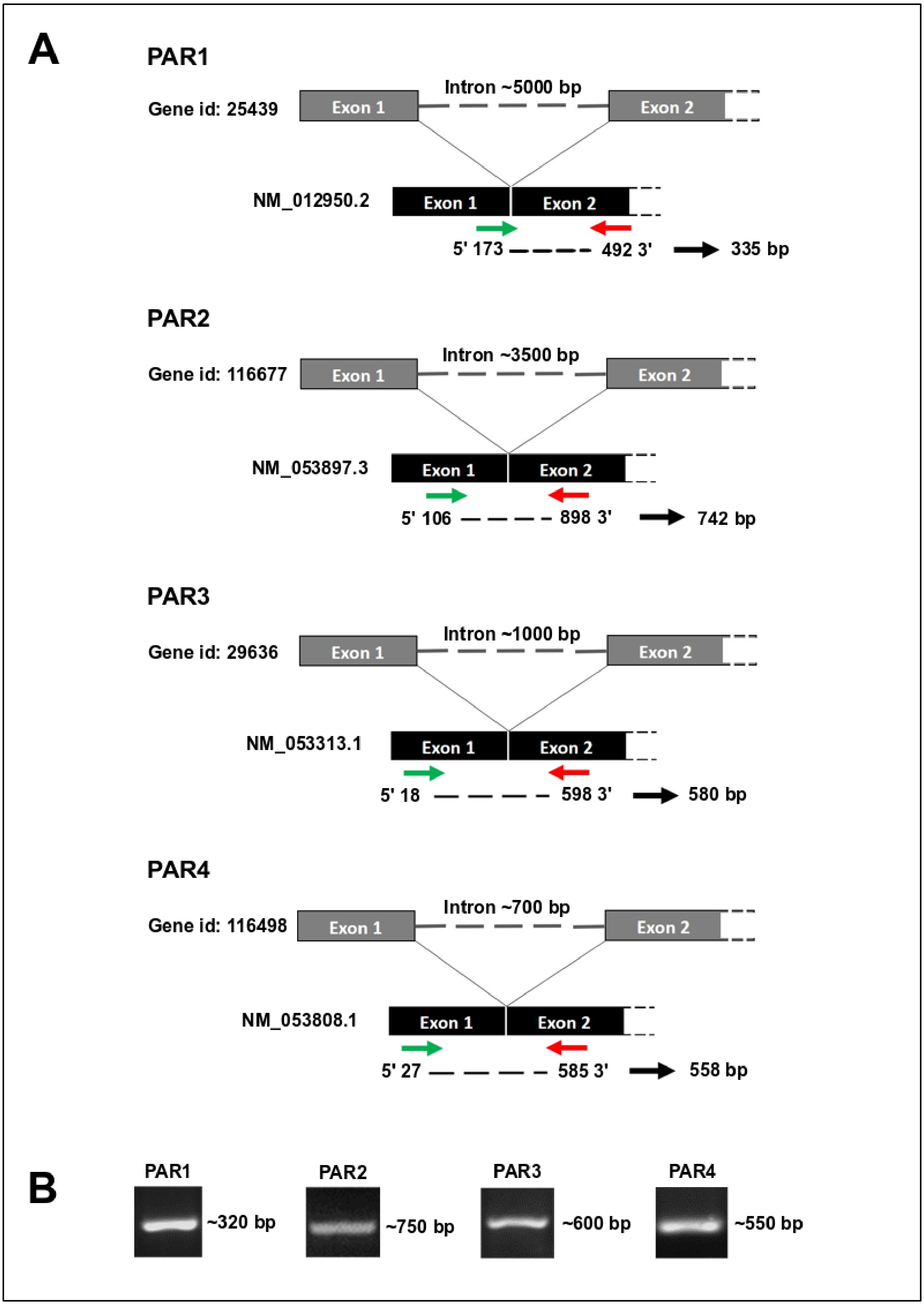
A
PAR1
Gene id: 25439
Exon 1
Intron ~5000 bp
Exon 2
NM_012950.2
Exon 1
Exon 2
5' 173
492 3'
335 bp
PAR2
Gene id: 116677
Exon 1
Intron ~3500 bp
Exon 2
NM_053897.3
Exon 1
Exon 2
5' 106
898 3'
742 bp
PAR3
Gene id: 29636
Exon 1
Intron ~1000 bp
Exon 2
NM_053313.1
Exon 1
Exon 2
5' 18
598 3'
580 bp
PAR4
Gene id: 116498
Exon 1
Intron ~700 bp
Exon 2
NM_053808.1
Exon 1
Exon 2
5' 27
585 3'
558 bp
B
PAR1
~320 bp
PAR2
~750 bp
PAR3
~600 bp
PAR4
~550 bp

Figure S1: All four PARs are expressed in GPNT cells.

(A) Schematic illustrating the predicted annealing position of the specific primers within each PAR gene (using NCBI reference genes as indicated). Note that each primer pair spans an intron. The small and much larger predicted amplification product sizes from cDNA and genomic DNA, respectively, are also noted. **(B)** RT-PCR products for PAR1-4 using the primer pairs described in panel (A). Observed sizes (in relation to a 50 bp maker) are noted on the right. Note that detected sizes corresponded to that of predicted cDNA but not genomic DNA products. The identity of all PCR products was further verified by the presence of unique restriction enzyme sites as well as DNA sequencing.
